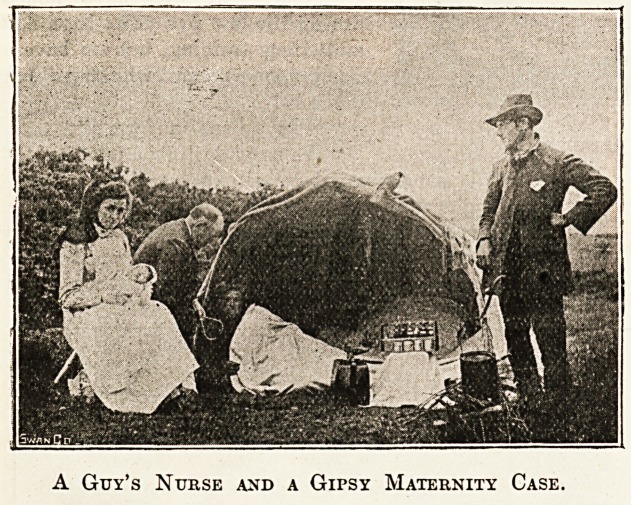# The Hospital. Nursing Section

**Published:** 1906-02-24

**Authors:** 


### The Hospital.

### Nursing Section.

Contributions for "The Hospital," should be addressed to the Editor, "The Hospital" Nursing Section, 28 & 29 Southampton Street, Strand, London, W.C.

No. 1,013.—Vol. XXXIX. SATURDAY, FEBRUARY 24, 1906.

### Notes on News from the Nursing World.

### THE RESIGNATION OF MISS MONK.

We greatly regret to announce the resignation of Miss Katharine H. Monk, sister matron of King's College Hospital. Miss Monk had desired that her resignation should not at present be made public, but an intimation having by some means got into print, we are authorised to state that this step has become necessary on account of the condition of her health, and that she is giving up nursing work and retiring into private life. Miss Monk, who has been matron of King's College Hospital since 1884, was previously ward sister and night sister in the same institution. She was trained at St. Bartholomew's Hospital, and has been engaged in nursing work for about thirty years, performing with characteristic earnestness and ability, in addition to her arduous duties at King's College Hospital, that of a member of the Committee of the Junius S. Morgan Benevolent Fund in connection with the Royal National Pension Fund, and a member of the Nursing Board of Queen Alexandra's Imperial Military Nursing Service. It is not too much to say that there is no matron in London who is more beloved by her staff, as there is certainly none who has done more to merit the confidence and affection of all who have been associated with her. How much her personality appeals even to outsiders was to our knowledge testified in a striking manner the other day, when a candidate for training was refused admission in consequence of her health, and was afterwards heard to remark: "I don't know when I have had such a disappointment! It is not that I feel not getting into King's so much, but that I would have given anything to have worked under such a woman as Miss Monk." The name of Miss Monk stands, in fact, for all that is best in nursing. Throughout the whole of her career her object has been not merely to turn out good nurses, but good women. This, indeed, was the primary reason why she instituted, more than fifteen years ago, a nine hours' day for nurses, which secured them four complete hours for rest and refreshment. As she herself said in an interview which appeared in our columns on April 29, 1899, she holds that a nurse ought not only to be a power in her work, but also in the world, and with that view she considers it essential that probationers should have sufficient leisure to keep in touch with interests beyond the walls of the institution. With delightful personal qualities, she combines the talents of a born organiser, and whether in respect to efficiency or to character, the nurses at King's College Hospital under her auspices have always enjoyed a high reputation. We are sure that Miss Monk, who is just now staying at Brighton, will carry with her in her retirement, the most cordial good wishes of those who know her by repute, and the loving sympathy of those who have the privilege of her acquaintance.

### THE POPULARITY OF PRIVATE NURSES.

The particulars given by the lady superintendent of Guy's Hospital Institution for Trained Nurses are a welcome proof of the popularity of private nurses, when they are efficiently trained, and conduct themselves satisfactorily in the homes of the patients. The staff attached to Guy's Institution has grown in twenty-five years from two to a hundred and ten, and on the day our Commissioner visited the headquarters in St. Thomas's Street, there were only four disengaged, while on the previous day there was but one. The lady superintendent is, therefore, abundantly justified in saying that the demand for nurses is very good. Moreover, the demand is not limited to England, but their services are in request in various parts of the world. We believe that if all nurses working on their own account were not only as well trained, but displayed the assiduity and the tact which seem to be possessed by those belonging to Guy's Institution, complaints on the part of the public would be few and far between. It is the badly trained, and still more, perhaps, the badly behaved, private nurse who is largely responsible for any prejudice against the calling she discredits.

### LADY ABERDEEN ON DISTRICT NURSING.

As we anticipated, the Countess of Aberdeen, wife of the new Lord-Lieutenant of Ireland, has lost no time in showing her profound sympathy for those who are engaged in promoting the work of nursing the sick poor in the sister island. At the twenty-ninth annual meeting of the friends and supporters of the St. Patrick's Nurses' Home in Dublin, her Excellency, having warmly praised the work done by that institution, proceeded to comment on the complaints that nurses in these days make too much of the professional side of their work. She thought that this might sometimes be the case with private and hospital nurses, but said that she was sure "that it is practically impossible for district nurses to be purely professional nurses, and to continue to be district nurses in the real sense of the word, because the demands made upon them cannot be met except by those who are embued with the spirit of devotion and a desire to consecrate themselves to the service of humanity." There is a great deal of force in this argument, which will be appreciated by many who read the experiences of district nurses which appear from time to time in our columns.

### THE POWER OF PENCE.

At the annual meeting of that excellent organisation, the Shoreditch and Bethnal Green District Nursing Association, which we report elsewhere, Miss Amy Hughes made an admirable practical suggestion for increasing the funds. In view of the urgent needs of the organisation, which last year had to reduce its staff for lack of means, Miss Hughes asked the men employed in the works and manufactories in the two boroughs to set aside a penny, or even a halfpenny, a week of their wages, place it in the hands of the foreman, and send the total at stated intervals to the association. The popularity of the nurses of the association in the boroughs which are the scene of their labours encourages the belief that the appeal of Miss Hughes will meet with a response, but she did well to cite the case of the colliers who, in a provincial town, support two nurses entirely, and have a balance in the bank, by the same method which she invited the working men in East London to adopt. We hope that the illustration she gave of the power of pence will have the effect desired, and that at the end of the present year the superintendent of the Shoreditch and Bethnal Green Association will be able to report that a new source of income has been tapped so successfully that she has been able to augment her staff.

### UNPROGRESSIVE GUARDIANS IN PADDINGTON.

It is disappointing that a Board of Guardians which has control over so well administered a Poor-law infirmary as that at Paddington, should fail to provide efficient superintendence in the sick wards of the workhouse under its charge. The Local Government Board has recently drawn the attention of the Paddington Board of Guardians to the increase in the number of deaths in the sick wards of the workhouse, and reminded the Guardians of their duty under the Nursing in Workhouses Order, 1897, to appoint a superintendent nurse. Instead of appointing a superintendent nurse, or a home sister, or a qualified nurse as assistant to the matron of the Paddington Workhouse, these unprogressive Guardians have refused to make the present sick wards, in nursing matters, as efficient as they ought to be. The Guardians plead that the excess of deaths in these wards is due to the limited accommodation at the Infirmary. This is Bumble in his worst form, for, if the infirmary wards are inadequate there is all the more reason why the poor patients who are thus compelled to remain in the sick wards of the workhouse should be provided with proper nursing care and supervision. Are the Paddington ratepayers wholly indifferent to the claims of the poor, or will they voice public sentiment in this matter and insist upon the Paddington Guardians appointing a superintendent nurse without further delay? We hope the Right Hon. John Burns will bring all the pressure that the Local Government Board can exercise to bear on these unprogressive Guardians.

### BULB GROWING FOR THE WARDS.

For some time the patients in the wards of a Lancashire hospital have day by day watched the growing to perfection of many beautiful spring flowers. In August last the nursing staff spent a few shillings on bulbs, such as the giant crocus, Roman hyacinth, and the daffodil (Tenby, Emperor, and Empress), which grow well in China bowls and look pretty at Christmastime and in the early spring when flowers are so rare. At the bottom of each bowl of peat was placed a piece of charcoal, to keep the earth fresh and free from unpleasant odour, and the bulbs when set were put into a dark room—not a cupboard—where they could have plenty of air but no light. For several weeks they were kept in the dark with little water, but when well rooted they were brought from their hiding-place and given as much light and sunshine as it was possible for them to have. When they had grown to a good size, and buds were showing well, they were placed in the wards. The patients began at once to take the greatest interest in them, noticing especially each day which bowl was put near to them, and claiming the flowers as they opened for their own. Each morning they observed the different buds grow and burst and open, and could even see the changes which had taken place in them during the night. The blooms have more than repaid the long time of waiting, for they have made the wards cheerful and gay, when few English flowers could be obtained. This experience of bulb growing has been a great pleasure to all, and the winter days will always be remembered for the brightness which the flowers brought to them.

### A PATHETIC INCIDENT.

The annual report of the Trained Nurses' Annuity Fund, of which Princess Christian is President, has been sent to us for notice. Prominence is naturally given to the loss sustained last year by the death of Lady Blomfield, the generous founder, by whose personal friends the Fund has been chiefly maintained until recently. It is now felt that an appeal must be made to the public to carry on the good work of helping deserving nurses who have spent the best part of their lives in the service of the sick. In no way is it sought by the Council to interfere with the efforts of those who are anxious to induce nurses to secure adequate pensions, but the object in view is to meet, to a limited extent, the needs of nurses who from various causes have been unable so to protect themselves. It is pleasant to learn that in 1905 nearly £40 was contributed by nurses, several of the annuitants themselves sending in their mite; and mention is made of the pathetic case of one on the waiting list so low down that it is very doubtful whether she will ever enjoy an annuity, who has been active in collecting for the Fund. Her example should act as a stimulus in other quarters. Every £500 subscribed provides an annuity of £17.

### NURSES AND FOREIGN APPOINTMENTS.

It seems necessary to caution nurses against accepting, at short notice, appointments in a distant country of which they know nothing. One of our readers was offered the other day a position as nurse matron of a new hospital in a remote part of Africa, but was informed that she was required to give up her present duties and start in a fortnight. To be prompt is a very good thing when promptness can be combined with wisdom, but the nurse in question appears, from her own account, to have entertained the offer without any clear idea whether the terms included food, attendance and laundry, or knowledge of the persons who are sending her. She is also ignorant of the nature of the climate, of the kind of outfit necessary, and other particulars which should be fully ascertained before serious attention is given to a proposal of the kind.

### NURSES INDIFFERENT TO THEIR INTERESTS.

The Council of the Royal Victorian Trained Nurses' Association have decided against the provision of additional accommodation for members, and we do not see that it was open to them to arrive at any other conclusion. Although 800 voting-papers were sent out, only 169 replies were received, and of these 27 were distinctly hostile, while few of the hundred absolutely in favour of engaging an additional room at a probable cost of £30 a year, signified their readiness to pay the extra subscription necessary to obtain sufficient funds for the purpose. The discouraging point, however, is, we think, the small number of answers received to the circulars asking for an expression of opinion. It is clear that in Australia, no less than in England, the indifference of nurses to matters affecting their own interests is an important factor in the situation.

### CUBICLES AT FAVERSHAM.

The Faversham Guardians at their last meeting had under consideration the question of the improvement of the nurses' quarters. The Chairman reported that the committee appointed for the purpose had inspected the nurses' quarters at Tonbridge and Dartford Unions, and they found that there were some very nice cubicles at Tonbridge, but with wooden partitions. They had not, however, it was added, learnt anything useful. Nevertheless, they appear to contemplate the erection of cubicles at Faversham, and a rough plan submitted to several of the Guardians is described as "a good idea." No plan which embraces cubicles for the sleeping accommodation of nurses can be a good idea, and we trust that the Faversham Guardians will not adopt a system discarded by all authorities who are concerned to provide nurses with the privacy to which they are entitled. We can understand the retention of cubicles where they already exist, but the deliberate introduction of them by reason of their supposed advantages is inexplicable.

### A NEW GLASGOW MATRON.

We are officially informed that Miss Helen Gregory Smith has been appointed matron of the Western Infirmary, Glasgow, in the place of Miss Shannon, who left the institution last week. Miss Smith, who has entered upon her duties, was trained in the school of which she now becomes the head, and was assistant matron for several years. She has lately been matron of Dumfries Royal Infirmary. The choice is in all respects admirable, and we are not surprised that the return of Miss Smith to the Western Infirmary has given general satisfaction.

### A NURSING SISTER FOR OBER-AMMERGAU.

On the afternoon of Wednesday, March 14, Miss Milner, who has been given, by the Duke of Westminster, the use of Grosvenor House for the purpose, will tell the story of Ober-Ammergau, with lime-light pictures and the assistance of some well-known artists. The proceeds will be divided between the Ober-Ammergau Cottage Hospital to complete the fund for the purpose of providing a nurse to live in the institution and attend the sick in their own homes, and the Hostel of St. Luke. It will be remembered that some little time ago Miss Milner contributed a sketch of the scheme to our columns, and we are glad to learn that in consequence she received several contributions to the movement for which she has done so much.

### RESIGNATION OF A MATRON.

Miss Ida M. Eastmond has resigned the matronship of the Wolverhampton and Staffordshire General Hospital, on her approaching marriage to Mr. Edmund Forster, M.A., late House Governor of the Wolverhampton General Hospital, and now Secretary of the Bradford Royal Infirmary. On leaving the hospital Miss Eastmond was presented with a handsome oak bureau and a set of carvers in case by the sisters and nurses, an egg stand by the domestic staff, and also some valuable gifts from friends connected with the hospital.

### CHOOSING AN ASSISTANT NURSE.

At their last meeting the Macclesfield Guardians selected an assistant nurse for the union infirmary, the choice being between three candidates who were recommended by the Visiting Committee. Of these three only one, however, was fully qualified, the experience of the other two being gained chiefly in mental asylums. Ultimately, the first was chosen by a majority of only four, although in addition to her three years' training she holds a midwifery certificate. Following her election, one of the Guardians intimated his intention to propose that probationers be trained under the auspices of the Board, but it is understood that opposition will be offered to the motion on the ground that it is more economical to engage nurses from other schools than to train them.

### SHORT ITEMS.

On Wednesday evening a concert was given at the Royal Horticultural Hall by the English Ladies' Orchestral Society, in aid of the Sick-pay Fund of the Colonial Nursing Association, which is much in need of assistance.—The cost of nurses' fees and travelling expenses during the late typhoid fever epidemic at Lincoln amounted to £3,313.—The Earl of Mansfield and Lord Cheylesmore have been appointed by Queen Alexandra to the Council of Queen Victoria's Jubilee Institute.—It was decided at a meeting held last week to establish a branch of the Essex Cottage Nursing Association at Walthamstow. Lady Rayleigh, who presided, said she was confident that the branch would be in full working order before long.

### The Nursing Outlook.

"From magnanimity, all fears above;
From nobler recompense, above applause,
Which owes to man's short outlook all its charm."

### THE METROPOLITAN MATRON.

The resignation of Miss Monk, after upwards of twenty years' invaluable service to King's College Hospital and to the wise development of skilled nursing in this country, is an event of more than passing importance. Miss Monk without doubt has proved herself to be one of the wisest, most knowledgeable, least prejudiced and distinctly valuable women workers who have held a Metropolitan matronship during the last quarter of a century. Her methods have been admirable throughout, and her aims of the highest. Her strength has lain in the quietness and confidence of her character, which has enabled her to bring the nursing department and school at King's College Hospital to the highest state of efficiency, whether it be regarded from the point of view of its output, or from that of the excellence of its organisation in detail. If the whole purpose and life work of the metropolitan matron is to be fixed upon the immediate interests of the particular hospital to which she may be attached, then Miss Monk's career is one which cannot fail to afford an example for others to follow. Miss Monk has, however, in recent years taken her share in the effort to reorganise army and navy nursing.

Is it good for the progress and development of nursing upon the highest lines in this country that all, or nearly all, the matrons of the great metropolitan hospitals should confine their energies and work entirely to the administration of the particular hospital with which they may be connected? Every matron has very responsible duties; to prove efficient she must be a woman of exceptional administrative ability, character and tact, and in the ordinary course of her work she must be brought into close contact with most of the more active workers in the nursing field. Hence she should possess an intimate knowledge of the requirements and needs of the profession as a whole. It has been held desirable in the past, and so has become the nearly universal practice, for each matron of a large hospital in London to decline to take an active public, or indeed any real part, so far as effective influence is concerned, in the solving of many questions which affect nursing as a whole, and the adequate training of probationers. Any general policy of the kind has been voiced almost entirely by the treasurer or chairman of a particular hospital, with a few members of the honorary medical staff who may have been personally interested in nursing. This system must be held responsible for many things which are unsatisfactory in regard to nursing matters in this country to-day. If, so long as she discharged her immediate duties to her own hospital with efficiency, every matron had been left free to exercise her own judgment, and to take such a course as she thought best in the interests of her profession, we have some confidence that the organisation of nursing as a whole would not have been so long ignored, and that the control of nursing affairs would have been to-day in the hands of a representative body of trained workers, to the great advantage of the public, the profession, the patients, and sick people generally. We are of opinion that the trustees and managers of the great hospitals in the United States have set an example in this matter which is worthy of the closest consideration of every hospital committee throughout the United Kingdom.

What is the American system? The larger hospitals are placed under the direct administrative control of a superintendent who is usually a member of the medical profession, though a few well administered American hospitals are in charge of laymen and women workers of experience. Every head of department reports to and acts in co-operation with the superintendent of each hospital, which secures the enforcement of a definite policy and the comfort and well-being of everybody connected with the institution. This system has worked admirably at Guy's Hospital, and at some of the larger Scottish hospitals especially, all of which may claim, with justice, to be amongst the best administered hospitals in the United Kingdom. In America, where there is a matron, she in fact discharges the duties which are usually relegated to an experienced housekeeper in this country, whilst the whole of the nursing arrangements and the training school are placed in charge of a lady superintendent. The lady superintendents of the American hospitals, whilst they work in harmonious co-operation with their superintendent and the boards of management, are free to take such a part in general nursing affairs outside the hospital as their experience and abilities may warrant. This system has created the American Society of Superintendents of Training Schools, which during the last twelve or fourteen years has united the nurses into one strong representative body, and has exercised a material influence upon the development of nursing education and kindred subjects in the United States.

The results produced by the American system have proved satisfactory. Has not the time arrived when every matron of a great hospital should be given her freedom, so that she may take her proper part in the development and improvement of her profession? She can thus alone acquire the position of authority which her knowledge and attainments may entitle her to occupy.

### The Care and Nursing of the Insane.

By Percy J. Baily, M.B., C.M.Edin., Medical Superintendent of Hanwell Asylum.

### I.—ANATOMY AND PHYSIOLOGY.

(*Continued from page 289.*)

### 5. The Circulatory System.

Under this heading we must consider: (1) The blood, which is a fluid tissue; (2) the heart; (3) the blood-vessels; (4) the lymph; (5) the lymphatic vessels and glands.

We have seen that our bodies are built up of an innumerable number of minute tissue elements called cells and that these cells may be regarded as having the same relation to the whole body as do individuals to a nation or community. Now, we as individuals have many wants, we must be supplied with the necessities of life—our bread and our tea and our sugar—we must be able to procure clothes, and when these are worn out we treat them as waste and get rid of them. In like manner the cells of our bodies must be supplied with the necessities of life, and the waste which is the result of the wear and tear of life must be carried away from them, even as our old clothes are borne off by the old clo' man. As we shall presently see, it is the blood which is the agent by whose means these necessities are carried to the cells and the waste borne away from them. We know from common experience that whenever we get a wound in any portion of the body, however small the wound may be, whether only a scratch or graze or the prick of a needle, the blood escapes. After the bleeding has stopped there still exudes from the wounded surface until healing is complete, a more or less watery fluid which in small wounds such as a graze, ultimately dries up and forms a scab. This latter fluid we may call (for our present purposes) lymph.

We must now learn something about the blood, what it is composed of and by what mechanical arrangements it is carried to the various parts of the body; what is the nature of the work it performs, and what are its relations to the various tissue elements.

### The Blood.

When we look at blood which has just been shed, without the aid of a microscope, we seem to be dealing with a simple red fluid. But if the blood be allowed to stand for a short time (two or three minutes) and then again examined, we find that it has undergone a very remarkable change; it is no longer fluid, it has, in fact, become transformed into a soft jelly—in other words, it has clotted or coagulated. If this clotted blood be allowed to remain for some time and then again examined, the clot will be found to have shrunk and to have become rather firmer, while at the same time an almost colourless (really pale straw coloured) fluid has been squeezed out of it.

Now let us put a drop of newly shed blood beneath a microscope and examine it. Our ideas about it will once more be changed. We find that it consists of two parts—namely, a fluid part which is almost colourless and a solid part consisting of numerous minute particles. The fluid part of the blood is called the blood plasma, while the particles which float in it are the blood corpuscles. After two or three minutes if we look through the microscope more carefully we shall be able to distinguish a number of minute hair-like filaments which form a network within whose meshes the corpuscles are entangled. These filaments are composed of fibrin, and the clotting or coagulation of the blood is due to its presence. So long as the blood is contained within the living and healthy blood-vessels this fibrin remains dissolved in the blood plasma and the blood remains fluid.

The corpuscles are of two kinds. The coloured or red, and the colourless or white. Fig. 8.

Fig. 8.—Blood Corpuscles.

1. Red. 2. White.

1. The red corpuscles are the more numerous (400 to 1). They are flattened circular discs like little biscuits, and are very soft and elastic. They are composed of a substance called protoplasm, which may be compared to the white of an egg, surrounded by a very delicate membrane or covering. They are so small that they can easily pass through the most minute vessels in the body, it would take about 3,200 of them set in a row with their margins touching to measure a line one inch long. In other words they are about $\frac{1}{3200}$th of an inch wide. When seen singly as with a microscope they appear to be of a pale yellow colour, but when massed together they give the blood its red colour. It is quite impossible to convey by words any correct impression of their vast numbers in the blood, but we may say that roughly speaking in one single drop of blood they are about as numerous as the whole of the inhabitants of the globe. Each of these corpuscles is really a modified cell. Their colour is due to the presence in them of a very complex chemical substance called hæmoglobin. Iron is one of the constituents of this body. This explains the reason why iron is prescribed in certain cases of anæmia; anæmia being a condition in which the hæmoglobin of the blood becomes deficient in quantity.

The function of these red corpuscles is to carry oxygen from the air to the tissues, as we shall see more particularly presently.

2. The white corpuscles are less definite in shape than the red and moreover their form varies from time to time. They are rather larger than the red corpuscles and are like them composed of protoplasm. They may be taken as typical examples of the simple cell. We need not here concern ourselves with their function.

The plasma of the blood is, as we have seen, an almost colourless fluid. It has a saltish taste. It is slightly "sticky." It holds various substances in solution including the foods which it gathers after these have been digested, from the stomach and intestines. It also contains various waste matters which are thrown into it as a result of the wear and tear of the tissues. In addition to these it contains the fibrin which, as we have seen, is the cause of the coagulation of the blood after it has been shed.

The power of coagulation which the blood possesses is the chief agent in bringing about the natural arrest of hæmorrhage.

(*To be continued.*)

### The Nurses' Clinic.

### MALTA FEVER.

Malta fever is not often met with in a general hospital, but these cases are most interesting to nurse and few better repay care and attention. The attacks of fever may be very short, lasting two, three, or even only one day, with intervals of days or weeks between each bout, or the fever may continue for several weeks, reducing the patient to a state of extreme exhaustion and emaciation. The convalescence in the latter cases is very slow. Relapses frequently occur, and complications arise, such as neuritis, gastric disorders and phthisis.

In most cases the onset is sudden—the patient may be feeling quite well or perhaps merely tired and listless, when he begins to feel shivery, and in from a few minutes to half an hour he has a severe rigor. The first step is to put him to bed between blankets, with two or three hot-water bottles, and as soon as possible a hot blanket should be put over him and well tucked in all round him.

If the doctor is not at hand, and if the patient's pulse is good, he may now be given phenacetin, grs. x, followed by a hot drink—hot lemon-water for choice, but barley-water, toast-water, or hot milk will do if he prefers them, the object being to make him perspire as soon as possible. Frequently the rigor is accompanied by a feeling of sickness, if not actual vomiting, which makes the task of inducing perspiration much slower, and also exhausts the patient. In these cases sips of boiling water may be tried—the hotter he is able to drink it the better. The rigor stage may last from half an hour to three or four hours, and then follows the fever stage. The patient becomes hot, restless, and extremely thirsty, and on taking the temperature it will be found to be between 103° and 105°, or even higher.

Generally speaking, this stage is quickly followed by profuse sweating. The nurse should have ready two warm blankets, a fresh warm flannel suit or shirt and two warm towels. In from ten minutes to half an hour the sweating will be diminished, and she must then remove the hot-water bottles and take off the patient's suit as rapidly as possible. He should be kept well covered with blankets and rubbed down with hot towels, a fresh warm blanket being rolled under him during the process. The second warm blanket can then be put over him and the clean suit put on.

Some people recommend sponging the patient down with warm water and vinegar, but as he is already considerably exhausted by the attack, and probably very sleepy, I have found it best to rub down with hot towels, sponging the hands and face only—this also minimises the risk of a chill during the process. The temperature should now be taken, and if it is down the nurse may safely give, in the absence of the doctor, quinine sulphate, grs. v, and a drink of warm milk. If the temperature is not below 100° the quinine had better not be given without the doctor's orders.

In some cases these three stages are not all present. In long-continued fevers the rigor is not so severe, the daily rise of temperature being marked by slight chilliness only; the hot stage is longer, and the sweating may be very slight or absent altogether, or else so profuse as to dangerously exhaust the patient. In long-continued cases the nurse should take the first opportunity to put in a long mackintosh to protect the mattress, as I have known the latter to be soaked through with perspiration where this precaution was neglected.

Of course, in these cases there is much wasting, and great care and attention are needed to keep the patient's skin in good condition. The shoulders, back, elbows, and heels must be attended to at least twice a day, and it is also a good plan to powder the patient all over after washing with a mixture of boric acid and starch powder, especially the chest and axillæ or any part where the constant sweating appears to irritate the skin.

The pains in the joints, which are such a frequent complication, are very severe. They will, of course, be treated by the doctor, and the nurse can only keep the affected parts warm and make the patient as comfortable as possible.

Heart failure is another complication which may occur, and the nurse must be very careful to keep the patient lying down and to avoid excitement and sudden movement.

The strength must be maintained by constant and regular feeds, the diet is as a rule generous in Malta fever, so that there ought not to be much difficulty in tempting the patient's appetite. During convalescence the temperature must be watched and the amount of sitting up and subsequently of exercise also must be regulated by the chart.

The slightest rise of temperature in the evening may be taken as a warning that too much has been done in the day. The patient should have as much fresh air as possible, and as soon as the doctor will allow it, he should be warmly wrapped up on a couch and wheeled out of doors—at first for only an hour or so, gradually increasing until he spends nearly the whole day out of doors. Cold weather does not seem to do any harm, but damp must be carefully guarded against.

### Incidents in a Nurse's Life.

### A NIGHT WITH A SUICIDAL PATIENT.

I was staff nurse in a large men's medical ward, and one night when I went on duty I noticed that No. 5 had a peculiar look in his eyes. He was a typhoid of the mild type, and so far we had had no trouble with him. He had been in the ward about a week; his temperature was not and had not been above 102°, and except when roused up for feeds he slept the whole night through. But now I perceived at once that his eyes had a strange glitter and that he watched me wherever I went. Nothing had been said about him in the report, except that his treatment was to be as usual. Once he called me up to him and said that he had stolen a large sum of money from his employer and that it was very much on his mind. I believed him then as there was no high temperature to cause delirium, and he seemed perfectly sane.

The night went on as usual. There were many bad cases to be attended to and No. 5 still remained wide awake.

INCIDENTS IN A NURSE'S LIFE—*continued.*

At midnight I went into the ward-kitchen for a meal, and gave my probationer special instructions to watch No. 5. No sooner, however, had I left the ward than I heard cries for help, and rushing in I found my probationer struggling with the man. He had waited for his opportunity, taken his medicine bottle from off the bracket above his head, quietly put it under him and smashed it, and then with a piece of the glass had cut his throat!

He was strong and powerful, but between us we got the glass away from him, and in the dark I could feel the warm blood oozing from the wound made. Fortunately no large artery or vein was severed; had he cut a little deeper nothing could have saved him. Night sister was soon on the spot, and then the house surgeon who sewed up the wound. There were several pieces of glass which he tried to hide in the draw-sheet, and no doubt if we had not thoroughly examined the bed he would have made another attempt.

He was full of repentance afterwards, and said he had done it because he was sure the doctors were trying to kill him, so that they could have his body for the post-mortem room. For the rest of the night I sat beside him, and even then towards early morning he nearly succeeded in getting out of bed. He gave one sudden spring, but I was on the alert, and with the help of the patient in the next bed, managed to force him down again. As soon as the morning work began sister sent me a special nurse, and that same day No. 5 was taken away to a lunatic asylum. Needless to say the affair was a great shock to us both, my poor probationer was fearfully nervous and shaky for many nights afterwards, and I can never be thankful enough that the attempted suicide was not successful.

### Private Nursing for a Quarter of a Century.

### INTERVIEW WITH THE LADY SUPERINTENDENT OF GUY'S HOSPITAL INSTITUTION FOR TRAINED NURSES. BY OUR COMMISSIONER.

At a time when the question of the attitude of the public towards private nurses is keenly discussed an authoritative account of one of the most famous institutions from which the latter are sent forth may be found exceptionally interesting. When I visited Guy's Hospital Institution for

Midwifery Pupils at Guy's Trained Nurses' Institution.

Trained Nurses at St. Thomas's Street the other day, Miss M. N. Oxford, who five years ago succeeded Miss Swift, now matron at Guy's Hospital, as lady superintendent, kindly suggested that I might like to see something of the quarters occupied by members of the staff when they are disengaged. As the great majority of the nurses are always away on duty, the three houses, which practically have been knocked into one, contain ample accommodation. There are three comfortable sitting-rooms, a nicely appointed little chapel, and nearly a score of bedrooms, though these include provision for the servants.

"The Institution," I said when we returned to the lady superintendent's office, "has, I think, been in existence a little more than a quarter of a century?"

"It was started," she rejoined, "in September 1884 with two nurses in one of these houses. By the end of the year they had increased to ten, and in 1891 a second house was added, the staff having by that date been augmented to fifty-two."

"When was the third house taken?"

"In 1900, when the number of nurses had risen to eighty-four. At the present moment it is 107, but it varies from 105 to 110. We consider the maximum number 110. These include the district maternity nurses."

"Are the whole of your nurses trained at Guy's Hospital?"

"Yes, and it is an advantage to know them two or three years before they come here. Of course, they are all fully trained."

### The Origin of the Institution.

"What was the origin of the institution?"

"I believe it was to have a sufficient staff of nurses available for the hospital to fall back upon. But as a matter of fact it is a great many years since any of them were available for the hospital. They only go to the hospital in order to see an operation and to keep up to date."

"I suppose that a specific number of probationers are always in training for you at the hospital?"

"We pay the hospital for the training of forty-two, who have to come in on the agreement made by us with the hospital."

"Perhaps you would tell me the terms of the agreement?"

"They are sent for training for three years, and come here for a year and a half afterwards. We have also nurses who come after the general training at Guy's to get their midwifery training here."

### Midwifery and District Work.

"The arrangement," continued Miss Oxford, "is to give them three months' midwifery training on condition that they work on the private staff for nine months at the rate of £25 a year. At one of the best special hospitals this would cost them not less than £40. But if they gain by the arrangement, so do we."

"How many nurses are usually engaged on district work?"

"Nine always. We commenced with two only a few years ago. They have midwifery training for three months, either paying in cash or giving their work for nine months like the others. I much prefer them to do this. It helps on the staff. At the end of twelve months they often take up work on their own account. The single year's agreement explains the large proportion of our nurses who are always coming and going. But, on the other hand, we have more than seventy on the permanent staff, some of whom have been here for eighteen or twenty years."

GUY'S DISTRICT NURSES: A GROUP IN THE GARDEN.

SALARIES AND BONUS.

"What salary do your nurses start with?"

"They begin with £25, and increase £5 a year up to £40. After the year in which they are paid £40 they commence to receive a share of the bonus."

"Are the profits of the institution divided among the nurses?"

"Entirely; no one else has anything out of it. The hospital does not receive a farthing, and the larger the profit the more the nurses get. It is true that it does not go into their pockets direct, but is invested for them in the Royal National Pension Fund after they have been attached to the institution for seven years. Since I have been here we have had nurses retire with a pension of £52."

"When are the nurses entitled to receive their annuities?"

"At the age of 55, and if the Institution remains as flourishing as it is at present, by the time a nurse is 50 she will have earned enough to bring her in £52 a year when she is 55. The bonus can be willed away by the nurse; it does not return to the institution."

"Then I conclude it is one of the rules that a nurse is not allowed a bonus after she is 50?"

"Only under quite exceptional circumstances. But she may continue nursing until she is 55."

SISTERS AT THE HOSPITAL.

"If a nurse leaves do you have her back again?"

"The custom is not to have a nurse back again, because the idea is for the nurses to stay. But here again exceptions are made under special circumstances. Since Miss Swift has been Matron of Guy's thirteen of our nurses have gone back to the hospital as sisters. This arrangement is much appreciated. Our nurses feel that they are not quite cut off from the hospital."

"Is your age of admission lower than that of the hospital?"

"No; our admission age is from 23 to 32. All our nurses have had children's training in the hospital, and many have had massage training, as well as experience in the light treatment."

"And midwifery?"

"Nearly all who have joined during the last five years have had midwifery training, but we have a good many nurses who have not taken it."

"What about uniform?"

"We provide it; in fact, except in their holidays our nurses have no expenses."

"How long are the holidays?"

"Every nurse has a calendar month each year. Nearly all the holidays are taken in the four summer months, especially July and August, when we are least busy. At the end of her fourth year in the institution each nurse has two months' holiday."

GUY'S NURSES AT WORK DURING A SMALL-POX EPIDEMIC.

WHERE THE NURSES GO.

"You do not seem to have many nurses here to-day?"

"There are only four in to-day, and yesterday I had but

### PRIVATE NURSING FOR A QUARTER OF A CENTURY—*continued.*

one. I see from my record that yesterday there were three absent on sick leave, four on holiday, and six engaged on district work, 94 being away on private cases; so that I think it may be said that the demand for our nurses is very good."

"It is not limited to England?"

"No, we send nurses to all parts of the world, including Australia, Central Africa, and America. There is an Association at Oporto which constantly has nurses from us, and they like going there. Two or three years ago I was asked to send one to Uganda. She was away for four months, out of which time she nursed the baby for 17 days. A great many nurses go abroad with their patients. One goes to Biarritz to-morrow. Not long ago I sent one to Athens to nurse a case of appendicitis."

"I suppose they go to all sorts of places at home?"

"Yes; they have been sent to Sandringham, Welbeck, Badminton, and many other historical seats. Then we keep a permanent nurse in three districts—Milton Abbas, Dorsetshire—where last summer our nurse's patient was a gipsy who lived in a tent; another at Westcott, Dorking, and the third at North Mimms, who is maintained at the expense of Mrs. Burns. I suppose that about half the cases

A GUY'S NURSE AND A GIPSY MATERNITY CASE.

are in town and half elsewhere. We nurse a great deal in places just a few miles out, like Sutton."

"The regulations, I conclude, are thoroughly understood on both sides."

"Each nurse is given a copy of the regulations and conditions when she goes to a case, and also a form, signed by the medical man in attendance and the patient when she returns. A candid expression of opinion as to her conduct is asked for in it. The outside time a nurse is expected to remain on one case is two months, but by express permission she may stay for four months. She is, however, permitted to write and inform me if at the end of two months she wishes to come away."

"Do you nurse all kinds of cases?"

"Yes. As to infectious cases, a nurse frequently disinfects the house, the patient, and herself also before she leaves. We have no trouble on that score. The number of applications for nurses averages more than twenty per week. The most slack time is when physicians and surgeons are away on holiday in the summer, and the most busy when there is an epidemic of influenza."

### THE DISTRICT.

"The official staff, I gather, is very small?"

"It is limited to the district midwife, the house nurse, and myself. Our nurses are exceedingly interested in the district work. We have about 1,300 midwifery cases in the year; half the staff at least have worked in it, and know how poor the people are. They collect money, old clothes, and linen for them, and make garments as well. I do not think that there is a nurse on the staff who has not done something to assist, although they have very little leisure. When they are at cases they are only supposed to have an hour off duty."

### Royal Maternity Charity of London.

THE annual meeting of the Royal Maternity Charity of London took place at the Mansion House on Tuesday, the Lord Mayor presiding. The Charity is one of the oldest of its kind in existence, having been started 150 years ago by a few benevolent City merchants in the East India Coffee House for the delivery of poor married women in their own homes. This year being its third jubilee, the Lord Mayor and some of the other speakers earnestly appealed to the general public for funds to carry on so deserving a work.

According to the programme of proceedings the President called upon the Secretary (Major Lionel B. Killick) to read the advertisement convening the meeting, and afterwards moved "That the minutes of the last annual general meeting, having been already duly published to the Governors in the last annual report, be taken as read, and signed by the President." The motion having been agreed to, the President called upon the Secretary to read the report of the committee to the Governors, and afterwards moved its adoption, which was agreed to.

The report gives some interesting details of the work. The total number of cases attended during the year was 2,804. The deaths of mothers numbered only three, that of infants 41. The staff at present numbers 72 doctors, 24 midwives, and 20 chemists. The midwives, some of whom speak German, Russian, and Yiddish, are all fully trained, certified by the Central Midwives Board, and, in accordance with the Midwives Act, are under its supervision and inspection. They have all recently been equipped with new nurses' bags of the latest pattern, and it is a proof of the efficiency of the nursing staff that not one complaint has been made by the inspectors as to the methods, equipment, or professional ability of any of the Charity's midwives.

Archdeacon Sinclair alluded to the extensive area of this work, which, though it emanates from a modest building in Finsbury Square, embraces the districts of Bethnal Green, Bow, Hoxton, Homerton, Haggerston, Kentish Town, Camden Town, Islington, and many others. He pointed out the contrast between the care and comfort and tenderness which surround the well-to-do in their confinement, and the crowded, dirty, unhealthy surroundings in which the great majority of London women have to go through the same suffering.

Mrs. Phillp dwelt on the great national importance of providing skilled attendance in confinement for that largest mass of the community, the labouring classes. We had learnt, she said, enough about physical deterioration to know that the future health and physique of every child depended to a great extent upon the care and skill bestowed upon it at birth and in the first weeks of its life. Another point, to which she called attention, was that the Charity is worked chiefly by men. It was a patent fact that the pain and peril of child-birth called forth the tenderest sympathies of men all the world over, and it was in the united efforts of men and women that the raising of humanity was accomplished.

The proceedings closed with a plea by Mr. C. Barham on behalf of the work of the Jewish midwives who, he said, are of inestimable comfort and help to a large number of cases in Whitechapel.

### The Bishop of Stepney and Miss Amy Hughes on Nursing in East London.

There was a very well-attended and representative gathering at the annual meeting of the Shoreditch and Bethnal Green District Association on Thursday last week. The chair was taken by the Bishop of Stepney, and after the report had been read by the Rector of Bethnal Green, the Chairman said that it was scarcely necessary to move the adoption of this report; their hearts had carried it as soon as their ears had heard it. He himself had never yet been attended by a skilled nurse, because he had never required any such assistance. He knew that there were many present who would say that the reward for such a boast would be that he would fall ill to-morrow. Well, if that did happen, he would come to this Association to be nursed. The chief feature in the report was the number of visits paid during 1905, which was 40,269. They all felt in the East End of London that the force they most liked to think about was the force of the nameless unrecorded acts of kindness that were always going on round about them. Many of the acts of kindness were mistaken, but these 40,269 visits were all acts of kindness which did nothing but good through and through. How keenly these visits were appreciated he could testify. He had never seen such a sight as the meeting called in this district some time ago to bid farewell to one of their nurses, when he saw numbers of men wiping their eyes and heard them say, "Wherever she goes I will never cease to thank God for sending her here." There was no one in the district who was doing more good and getting more thanks than a nurse. He knew nurses objected to being "covered with butter from the platform," as they complained was the case at these meetings. They did their work because they liked it, because it was their duty, and because they wanted to help others; but they did not want to be thanked for doing it. Yet he thought that nurses scarcely realised what a debt of gratitude the representatives of religion owed them. When they looked back to the example of Christ they saw that there were always two sides to His work; the work for the souls was always combined with work for the bodies. Things had changed since then, and now clergy had to turn to doctors and nurses to do half their work for them. One clause in the report gave him pain, and that was where it stated that one staff nurse had been withdrawn for lack of financial support. There were, in his opinion, a good many people who could be taken out of the district and leave much less regret behind them than a single nurse. He hoped that all would do what they could to help the Association and enable them to maintain a full staff, and thus do their work with even greater efficiency in the future than they had done in the past.

The adoption of the report having been seconded by the Rev. H. Atkinson,

Miss Amy Hughes, General Superintendent of the Queen Victoria Jubilee Institute, who was received with much applause, observed that she quite agreed with the Bishop that nurses did not want to be praised or thanked; their work was a privilege to them. Therefore she was not going to say a single word about what the nurses had done, or were doing, for they all knew it. But she wanted to talk about something not half so interesting, yet even more important, and that was, How were they going to keep the home going, and where were they going to get the money from? This was what Miss Böge, the Superintendent of the Association, had asked her to speak about. It was often mistakenly supposed that the Jubilee Institute, having had large sums of money given to it, could support these Associations. But they were bound down by their charter only to spend their money in a certain way—*i.e.* upon the training of Queen's nurses. This Association was one of the largest training homes in London, and it therefore received in the money paid to the Committee for the training of their probationers a very fair proportion of the sum contributed to the central funds. The Jubilee Institute could not do more, having many calls upon its funds; it was always wanting more money because the country was always wanting more Queen's nurses. They must have noticed how often the nurses of this Association changed; that was because they went to carry out in other parts of the country the lessons they had learnt whilst in the Home. The people of Shoreditch and Bethnal Green must do something more to keep the Home going and relieve the Committee of so much worry and anxiety. Committees hardly ever got thanked at meetings; they were looked upon as the machine for grinding out money. Large sums were not expected; it was the small sums that mounted up, and there must be many who could afford 2s. 6d. or 2s. a year. She had a practical suggestion to offer them. She understood that there were a large number of works and manufactories in the two boroughs. Now it would be a tremendous help to this Association if all the men in the works combined together to set aside ½d. off their wages each, or, if they were very rich, 1d., and let it be put in the hands of some responsible person (such as the foreman), and they would be surprised at the pounds that would come in. She was sure that Shoreditch and Bethnal Green did not want to be beaten by colliers and miners; but the colliers of a comparatively small place followed this plan and supported two nurses entirely, and had a balance in the bank. She hoped that all the women present would tell their husbands, fathers, and brothers of this suggestion. At the Institute they were very proud of the Shoreditch Home and its nurses, and she thanked the Committee and subscribers on behalf of the Institute for training their nurses. The nurses not only ministered to the sick, but helped to make the homes a little healthier and brighter, and, above all, they taught mothers how to bring up their children. They were realising the object with which Queen Victoria had founded the Institute; they were fulfilling the function of health missioners and were a real influence for good amongst the people of our land.

Dr. Lewis Smith, of the London Hospital, said that a nurse must be three things: First, a woman and all that that entailed; secondly, a gentlewoman; and, thirdly, a trained woman. The nurses taught the people in the East End that windows were things to be opened, that babies were things to be looked after, and that dirt was anything in the wrong place. The nurses by their work among the poor were rendering it impossible that there should ever be a French Revolution in England, because the kindness shown in these 40,269 visits taught the people that there were those who cared for them and wanted to help them.

### To Nurses.

We invite contributions from any of our readers, and shall be glad to pay for "Notes on News from the Nursing World," "Incidents in a Nurse's Life," or for articles describing nursing experiences at home or abroad dealing with any nursing question from an original point of view, according to length. The minimum payment is 5s. Contributions on topical subjects are specially welcome. Notices of appointments, letters, entertainments, presentations, and deaths are not paid for, but we are always glad to receive them. All rejected manuscripts are returned in due course, and all payments for manuscripts used are made as early as possible after the beginning of each quarter.

### Clapham School of Midwifery.

The second open examination of the Clapham School of Midwifery was held at the Clapham Maternity Hospital on Saturday, February 9. It consisted of three parts—the writing of a paper occupying from 9 to 12 o'clock, followed at 3 P.M. by a viva voce, and finally by a clinical examination in the wards. It is found to be of advantage to the candidates to have the whole examination completed in one day; and the fatigue of this plan was minimised by the fact that the candidates were supplied with hot milk and biscuits in the middle of writing the paper in the morning, and with tea in the afternoon before the clinical examination, as well as having three hours for rest and food between the examinations. The Clapham examination is intended primarily for midwifery pupils who do not require to be registered as midwives, but desire nevertheless to possess some proof of having been trained in midwifery. It is also intended for candidates for the examination of the Central Midwives Board who, in addition to becoming registered midwives, wish to be able to produce a certificate showing at which school of midwifery they were trained. Nineteen candidates presented themselves. Of these four passed "with distinction" (*i.e.*, with above four-fifths of the marks); eleven "passed"; and four failed to satisfy the examiners.

### A Wet Day Episode.

BY A QUEEN'S NURSE.

A murky morning in a seaport town. It was raining dismally, and I was weary—very, very weary, both in mind and body. The walking long distances carrying a "district bag," to which I had been unaccustomed; the long streets, all so much alike, but all equally dirty; the strangeness of the work and surroundings had tired the body; whilst the painful sights and sounds, the poverty and distress which I seemed to have no power to relieve; the wickedness and drunkenness which I could not help seeing had depressed me. The pat-patter of the rain went steadily on, and another pat-pat seemed to accompany it. I heard without realising, my mind was musing on district nurses and their work among the poor. "What are they among so many?" the little band of workers and the great multitude waiting to be helped. Suddenly the soft flap-flap I had heard through my musings ceased close to me. I lifted my eyes, and, strange though the apparition was, I felt no surprise. I saw a tall figure clad in Eastern dress. With his long white robe, crimson sash, turbanned head, his dusky skin, long beard, and bare feet he looked like one of the "wise men from the East." Just pausing an instant, he raised the back of his hand to his forehead with Oriental politeness, made a profound salaam, and in a tone of deep feeling he said simply, "God bless you, Sister." The little incident was over in less time than it takes to tell it. I had but time to smile and bow a little acknowledgment of his salute, and he had passed on his way as silently as he had approached, and I passed on mine, touched and pleased. Only a word and a look of trust and reverence from a stranger from a strange land; but it changed the whole day for me, and sent me on my way, cheered myself, to try to carry cheer and comfort to others.

And the moral? Some nurse—some "sister"—had been good to this stranger; my sister and yours, whoever she was; one of our great sisterhood. For the good she had done him, he had evidently ever after revered any whom he saw clad in garb like hers. And I too echo "God bless you, Sister"; whoever you were, I thank you. Through you was I "strengthened" that wet and dreary morning; and I write it here with the hope that it will help other nurses—my sisters. all of them—to realise how far our influence—and whether we wish it or not—must carry, either for good or evil.

### Everybody's Opinion.

**[Correspondence on all subjects is invited, but we cannot in any way be responsible for the opinions expressed by our correspondents. No communication can be entertained if the name and address of the correspondent are not given as a guarantee of good faith, but not necessarily for publication. All correspondents should write on one side of the paper only.]**

### BLUFF AND BLUSTER.

"B. Kent" writes: I entirely agree with Nurse Lunn in her views upon this subject. The spirit of push and self-assertion is unbecoming in all women, more especially in members of a woman's highest calling. I can also join my testimony with hers concerning the doctors. I hold the medical profession in the very highest esteem; and from the individual members of it whom I have met, and under whom I have worked, during my thirteen years' experience as a trained nurse, I have received—with scarcely an exception—unfailing courtesy, consideration, and generosity of spirit, and in saying this I voice the opinion of my colleagues. If a nurse cannot "get on" with the doctor I venture to say that is more likely to be her fault than his. They are always ready to recognise and sympathise with the difficulties and responsibilities of a nurse's duties. They cheer and encourage us if the burden seems a little heavy at times, and they never fail to give us praise when they think we deserve it; sometimes even turning from themselves the credit and thanks of the patient—most justly due to them—and giving it to us. They treat us as co-workers with themselves, and they are our best friends. I have always appreciated this generous spirit of freemasonry that doctors show to nurses. But what about the rest of the world? Are we not enormously indebted to them? Can we adequately compute the good they do? By their skill and knowledge we know, of course, that humanly speaking they save many lives, but I think no one knows as well as nurses how self-sacrificing they are, how sympathetic and gentle. My loyalty to the doctor, as a nurse, is, I think, quite as great as my loyalty to my King as his subject. "Honour a physician with the honour due unto him."

### OVERWORKED PROBATIONER NURSES.

"Fairplay" writes: I should esteem it a favour if you could spare me a little space in your valuable paper to publish the following facts *re* long hours and no off duty for nurses. For the past year I have been staff nurse in a small isolation hospital, the staff being as follows: Matron, one staff nurse, one assistant nurse, and a probationer. The probationer's age is nineteen. The Medical Superintendent is non-resident. At the time of my appointment I was told that I should have as clear off duty one half-day from 2 P.M. till 10 P.M., one evening from 6 P.M. till 10 P.M., alternate Sundays 4 P.M. till 10 P.M. No holidays were given unless by arrangement at the convenience of the Medical Superintendent and Matron. As there are no wardmaids the nurses have the entire cleaning of the blocks to do, including grates, waxing and polishing floors and windows, as well as the nursing of patients. There are in each block two wards, one kitchen corridor, bath-rooms and sink-rooms. When two blocks are opened up for patients the probationer is on duty in the scarlet fever block, and she has to sleep in the ward and attend to the patients during the night, as well as having her usual work to do during the day. Her off-duty time is *nil*, with the rare exception of an hour perhaps twice or three times in two weeks. The staff nurse and assistant run the diphtheria block, and for many weeks I was called at 1.30 P.M. to take duty for the day nurse on her half-days and evenings, being on duty till the following morning 8 A.M. The work becoming heavier, as we were taking in cases of puerperal fever into diphtheria wards, and also an increase of diphtheria cases, institution nurses had to be called in to assist. Notwithstanding this, when I insisted on my off-duty time, and stated that nurses could not work the hours with heavy cases, I was told that we could not expect to have afternoons and evenings, but that we could go out after 8.30 p.m., and consider that our off duty, as the doctor did not wish the institution nurses to be called for extra duty, and they had objected to being asked to do so. I merely wish this to be put before the public to show that the rider added by the Coroner at a Dublin inquest was extremely applicable in the case of small institutions.

### NIGHT NURSES' HOURS.

"One Just Going on Night Duty" writes: I read with great interest and pleasure the opinions of "A Provincial Nurse," "A Lover of Justice," and "A Day Sister Reformer" in your issue of February 17th. As regards "A Provincial Nurse" I thoroughly endorse every word she says. Why should the nursing profession be so overworked? Surely it is very false economy, and why should it be such a crime for a nurse to complain of not feeling well? Surely a fresh, energetic woman is more fitted to tend the sick than a weary and worn one, and yet no nurse is ever thought good for anything unless she has used up her last atom of strength. And what is a nurse nearly always told when she complains of not feeling well? That she is hysterical; and she certainly is not encouraged to complain again. In my opinion nursing is so arduous that it requires the best of health to do the work as it should be done, and the smallest ailment, whether it be a bad cold, a headache, or a bad finger, should be enough to send a nurse off duty until she is quite well again. As regards "A Lover of Justice," I cordially agree with her, and in the fast approaching time when I shall be on night duty myself I shall feel that I have her sympathy. The life on night duty is certainly one of complete self-denial if the nurse does her duty, or else it is most demoralising. I have only one thing to say in response to "A Reforming Day Sister." I wish that when she becomes a Matron it may be my happy lot to work for her.

"B. M. A. F." writes: In reply to "A. M. M. B.," who does not appear to be of the opinion that the hours of night duty are too long, she seems to overlook the fact that not only is every turn the reverse way of nature, but, as the reforming day sister wisely states, how imperfect is the sleep the night nurse gets, a few cases excepted. I, for one, know what it is to go to bed at the regulation time, which is usually 12 A.M., and have heard every strike of the clock till the maid has called me for duty, because it is so unnatural to sleep in the day. And perhaps when just dozing off some thoughtless person trips by your door, singing, talking, or walking as heavily as may be. Naturally the nurse is not fit for her long watch. Even granting that, as some one said a week or so back, the first three hours after the night nurse comes on there is little or nothing wanted, and that for the last three hours the day nurses are on, I would ask, would it be possible to stand the strain of the long twelve hours and do as much work as is done in the day? "A. M. M. B." does not seem to think night duty more detrimental than day duty to a nurse's health. For my part I think that there could not be a comparison drawn between the two duties. The 24 hours are divided up between the two sets of nurses. The day nurse gets her time varied in this way: she goes off two hours and a half after she comes on, to what is called dress time, for half an hour; dinner and tea the same, and in most hospitals she has two hours off duty every day. The night nurse comes on for her twelve hours. She not only is alone all night, with the exception of seeing the night sister on her round for ten minutes, saying nothing of the many ghastly things which seem to happen in the night; but she has to take her food in the immediate vicinity of her patients, breathing the same air, which is unhealthy. Certainly she can go into the ward kitchen; but how far is that generally from the ward? I am in a phthisical ward and can hear my patients coughing and expectorating, and have only to raise my eyes in order to see them, thus keeping the work for ever before me. No nurse feels like eating all she should do under these circumstances. There are some hospitals where the nurse goes off for her food, but very few. Of course, some one must do night duty, but if the hours cannot be lessened a way might be made for their relief for food taking, and thus might be saved many a breakdown, caused through not taking food properly and the extra strain of night duty.

"M. R." writes: I think that "A. M. M. B.," who writes on this subject in the issue of February 10, must have extremely small ideas of what a night nurse has to do during the twelve hours of unbroken duty when she says that "the first three hours there is hardly anything to do," and that a great part of their work is simply to watch sleeping patients or listen for calls. I have done a good deal of night duty, but I never found time for sewing and very little for reading, only just snatches of news in the dailies. When I am on duty I cannot lose myself in a book, or I might fail to see a change in a patient or forget a feed or a dose, or miss giving a special medicine. "A. M. M. B." does not appear to think of foments, poultices, medicines, pulses, temperature, respirations, feeds, drinks, changing ice-bags, ice to suck, dressings to be attended to, and many other things which a night nurse has to do, fetching and preparing everything, and perhaps three or four four-hourly hypodermic injections, with one or two delirious cases getting out of bed, and occasionally a death. I have never been without cases that needed constant watching and care during the whole of the twelve hours, which I cannot say seems long because there is so much to do. Then the morning's work—attending to helpless ones, taking temperature, pulse, respiration, writing report of cases, washing several patients, and making beds, etc.—is enough to keep one fully occupied. Sleeping quarters should always be apart from the day nurses' bedrooms; also a strip of matting should be put down along the corridors, so that the constant going up and down of the women workers or dormitory maids could not be quite so plainly heard when the nurse is dead tired for want of sleep and finds herself being constantly awakened from sleep. Then, again, the night nurse does not get the long hours of quiet unbroken sleep which the day nurse enjoys, and no matter how fresh she tries to keep the air of the ward, one knows it is impossible for the nurse to get as much fresh air herself as the day nurse can. The lonely little meal prepared by the night nurse to be taken in a kitchen surrounded by different utensils used by the patients is not always eaten with relish. Often it is difficult to find time to prepare even a cup of tea, and three or four attempts are made before one is successful. If these minor comforts, if I may so call them, of night nurses were studied more, they would come off duty more cheerful and less tired, and be able to stand the strain longer and better.

### AN ADVENTURESS AS NURSE.

"Truthful" writes: In last week's issue of The Hospital a paragraph was inserted concerning "An Adventuress as a Nurse." Why that talented lady directed her attentions to the nursing profession it is hard to say. One wonders if she thought that nurses were more easily duped than any other class of people; at any rate, she seems to have gained admission to more than one good institution. By what means she did so remains unknown; whether she had good testimonials or a certificate is not mentioned. The honourable name she bore, or assumed, must have placed her above suspicion and protected her from further inquiry into her antecedents, and forcibly calls to mind the old adage, "There's nothing in a name." I agree with the moral, that a matron cannot be too careful in choosing her nurses. Even with most excellent testimonials, good education, and respectable family the candidate may not be a suitable nurse. That remains to be proved. The personal qualities of the nurse should be studied, not altogether those of her family. It is a regrettable fact that public opinion of nurses in the present day is not in their favour. Is there a cause? If so, what is it? Are the training schools turning out an inferior article labelled "trained nurse," or is there too little attention paid to the moral qualities and all to the physical? What qualifications should we look for in the suitable nurse? Opinions may differ, but my opinion is that a nurse should be amiable and patient with those under her charge, whether assistant nurses or patients, conscientious in her work, with an accurate attention to details, and, above all, strictly truthful. It seems superfluous to mention the latter qualification, but I regret to say that it is sometimes wanting. Perhaps I am not the only one—in fact I venture to say that I am not the only one—who has found something done, or undone, as it should not be, and could not find the person who was at fault. No one knows; no one did it. Serious friction is caused in this way; everyone lies under suspicion, and why? Because the offender will not tell the truth. The innocent resent suspicion, the guilty escape just reprimands. So the fact remains that truthfulness is the most important moral quality in a nurse. Time and observation are the only true tests of character. The most careful up-bringing is not a sufficient guarantee that a nurse will go through her training and remain the same character she was at the outset. Hospital life either ennobles or demoralises us—usually, I am pleased to say, the former, sometimes the latter.

### THE BABY SHOW AT FULHAM.

THE MATRON of St. Clement's Maternity Home and Nursing Institution writes: In connection with the "Baby Show" which was held at the Fulham Town Hall on the 13th inst., I find on inquiring of the mothers of the prize babies since that 41 are being "nursed by the mothers," five are being fed on cow's milk and barley water (prepared according to the directions that were given to the mothers whilst we were attending them), one was on Nestlé's milk, and one on Davis' malted food mixed with cow's milk. Neither of the judges asked about the feeding, in order that an unbiased opinion might be formed. So I think it is a matter of congratulation that so many nursing mothers gained the prizes, and that cow's milk and barley water stood next. I shall be very pleased if you will publish this statement, as it is of public interest. I should like to add that several of the "nursing mothers" go out to work, but that they arrange for someone to bring the baby to them, or for the mothers to run in home.

### SODA WATER AND BURNS.

"C. A. H." writes: My opinion of strong soda water for burns or scalds is a very good one, as I know from personal experience. When quite a young girl I scalded my hand and arm very badly with boiling fat. An old cook who was in our service at that time came to the rescue before anyone else could interfere. She got some very strong soda water as hot as could be borne, and held my hand and arm in this water for nearly ten minutes. Afterwards a dressing of olive oil only was applied. Boracic lotion was used for removing the dressings, but in a very short time the soreness had quite disappeared, without leaving the faintest scar.

### Where to Go.

A CONCERT and dramatic entertainment in aid of the benevolent fund (out-patients') of the Birmingham and Midland Hospital for Women, Out-patient Department of the hospital, Upper Priory, Birmingham, Saturday, February 24th, 1906, at 7.30 p.m.

### Appointments.

[No charge is made for announcements under this head, and we are always glad to receive and publish appointments. The information, to insure accuracy, should be sent from the nurses themselves, and we cannot undertake to correct official announcements which may happen to be inaccurate. It is essential that in all cases the school of training should be given.]

CHELSEA HOSPITAL FOR WOMEN.—Miss E. C. Laurence has been appointed Matron. She was trained at Guy's Hospital, where she was afterwards Assistant Matron; and she has been Matron of Victoria Hospital, Keighley.

CHESTERFIELD AND NORTH DERBYSHIRE HOSPITAL.—Miss A. E. E. Stallwood has been appointed sister. She was trained at the Royal Infirmary, Hull, and has since been assistant nurse at the Northern Fever Hospital, London, and holiday sister at the Royal Infirmary, Hull. She has also been attached to the private staff of the Hull Infirmary, and has been in charge of the Isolation Hospital, Market Rasen.

CITY OF LONDON HOSPITAL FOR DISEASES OF THE CHEST.—Miss Grace Buchanan has been appointed staff nurse. She was trained at Kensington Poor-law Infirmary.

HASTINGS BOROUGH SANATORIUM.—Miss Margaret Cotter has been appointed charge nurse. She was trained at the Royal Berkshire Hospital, Reading, and the City Hospital, Aberdeen.

IPSWICH UNION INFIRMARY.—Miss Mary F. White has been appointed superintendent nurse. She was trained at St. George's Hospital, Fulham Road, London, where she was afterwards sister, and has had experience in fever nursing at the South Western Fever Hospital. She has also been charge nurse at Romford Union Infirmary, and superintendent nurse at Aylesham, Norfolk.

QUEEN VICTORIA'S JUBILEE INSTITUTE FOR NURSES.—Miss E. Myers has been appointed superintendent of the Sheffield District Nursing Association, affiliated to the Queen Victoria's Jubilee Institute for Nurses. She was appointed a Queen's nurse in January 1894, and since October 1898 has held the post of assistant district superintendent at the Scottish Central District Training Home in Edinburgh. Miss Helen Clayton has been appointed superintendent of the Kensington District Nursing Association, affiliated to the Queen Victoria's Jubilee Institute for Nurses. She was appointed a Queen's nurse in January 1896, and has been superintendent of the Queen's Nurses' Homes in Darlington and Southampton. Miss Amelia Holbrook has been appointed superintendent of the Lincolnshire Nursing Association, in affiliation with the Queen Victoria's Jubilee Institute for Nurses. She was appointed a Queen's nurse in January 1899, and has since held various posts under the Institute. She is at present superintendent of the Stockport Nursing Association.

ROYAL EAR HOSPITAL, SOHO, LONDON.—Miss M. H. Weale has been appointed a staff nurse. She was trained at Croydon Union Infirmary.

ST. NICHOLAS CRIPPLES' HOME, BYFLEET, SURREY.—Miss A. Isabel Dalzell has been appointed matron. She was trained at the Queen's Hospital, Birmingham, was afterwards sister at the Royal Orthopædic and Spinal Hospital, Birmingham, took holiday duty for four months at the Birmingham and Midland Hospital for Women, was matron of a private hospital at Birmingham, for two and a half years, matron of the Hospital, Castle Douglas, N.B., for two years, and matron of the Rosehill Children's Hospital, Torquay, for three years. Between her appointments she has twice acted as temporary matron of the Royal Infirmary, Middlesbrough, also twice at the Rosehill Children's Hospital, and has done a good deal of private nursing.

### A Book and its Story.

### AN AUSTRALIAN HEROINE.*

Curtis Yorke's new book sustains her reputation as a writer whose stories are always charming and distinguished by bright and natural dialogue, clever characterisation, sympathy, and insight, with the rare gift of making the story tell itself, although in the present novel it is not the story that is the most absorbing point in the book. The characters in it are so skilfully drawn, so true to nature, so living that one's attention is naturally concentrated on their development.

Kitty Revelstone—"irresponsible Kitty"—is a well-defined sketch of a consistently inconsistent personality. Half sprite, half woman, wholly selfish. Her beauty is of the type that usually accompanies this irresponsible combination. Self-confident and fearless, with a freedom in manners and speech natural to a girl brought up in Australia, she forms a striking contrast to her elder sister Winifred, who is the real heroine of the story, and whose finer nature has a depth and natural reserve wholly absent from that of irresponsible Kitty. The book begins with the death of their mother. Kitty was her mother's favourite child, and her anxiety in leaving them was all for her, not Winifred's, future. She is asking Winifred to devote herself absolutely to the care of Kitty when she is gone. "I want you to promise to make Kitty's happiness come before everything, that, if need be, you will put yourself and your feelings entirely out of the question, as far as she is concerned." The girl raised her deep, dark eyes to her mother's. "Mother, is that quite fair?" she said slowly. "Promise," insisted the invalid inexorably. "Promise. Oh, Winnie! I thought you loved me!" The rarely used diminutive touched the girl strangely. "I do," she cried passionately; "you know I do." "Then promise. It may be the last thing I shall ever ask of you." "I promise," the girl said almost inaudibly. "I shall keep you to your promise remember," said Mrs. Revelstone, after a silence, in a harsh tone. "Mother, have you no love for me? Is it to be always Kitty, only Kitty?" the girl said with a break in her voice. "My dear, of course I love you too. . . . But Kitty is so gay and sweet and lovely. And she is so frail and delicate. Trouble or care or sorrow would kill her, my little Kitty." As she spoke Kitty's voice came to them across the garden, rising and falling in a lilting coon song. The mother raised her hand over her eyes, and two heavy tears rolled down her faded cheeks.

"Don't, don't, dear mother," whispered Winifred. "You may trust me." And that night when she was left motherless, Winifred knelt at her open window and found comfort in looking at the solemn stars. "We know, we know," they seemed to say, and in some vague way the forlorn young heart seemed comforted. From her childhood Kitty had always been first with her parents. Her handsome light-hearted father would give some passing caress to Winifred and then invariably ask, "Where's Kitty? Where's my darling little girl?" Winifred was not jealous. It was something deeper than that. She loved Kitty dearly. But few of us find a perpetual extinguisher exhilarating. And it is difficult to be always contented with a back seat in life's great show, even when we are old. And Winifred was very young.

The two girls, barely more than children, of fifteen and sixteen were left badly off, and at their mother's death they received an invitation from an aunt who was a stranger to them to go to London to stay with her. The invitation was accepted. And, in company with the faithful Scotch servant Tryphena and their dog Bounce, they started on their journey. Kitty was filled with expectations of the delights that life in London would bring to them. "And who can tell what lovely things may happen to us there?" she said. "Eh, lassie, happenings is in the hand of the Lord," said Tryphena, in her strong Scots accent, "and what He orders will come to you, whether it's in London or Australia, or the parish of Lanark, where I was born more than sixty years ago, when I took service with your mother, and had buried two sweethearts and said no to a third. For I was well favoured in those days, though you mightn't think it to look at me now." The last day had come, and they were on board ship, looking towards land. Kitty was a good deal given to crying when her feelings were worked up in any way. Winifred, on the contrary, seldom shed tears. As she told Kitty once, "she ached too much to cry." But all the same, there was a lump in her throat to-day, for, after all, Australia was the only home they had ever known, and England's advantages were still problematical.

Among the passengers was Sir Basil Derrick, an English baronet of thirty-five, who was returning home after an inspection of some Australian property which required his attention. He was a man who is described as being stern looking and sparely built, who women as a rule found worth exploiting, but upon whom, so far, no woman had made a lasting impression. As a rule, he was rather bored than otherwise by them. And, as an actual fact, he had never in all his thirty-five years had the faintest approach to a genuine love affair. There had been a brief engagement to the daughter of an impecunious friend of his family, a handsome, heartless woman, whom he found to be utterly unworthy of any honest man's attentions, and at the end of a month, finding this was so, he severed the engagement, and she married a friend of his who died shortly afterwards. She was on board the same ship and was returning to England. He avoided her on every possible occasion. But she was still as much in love with him as it was possible for her to be with any man. Sir Basil had struck up an acquaintance with the two Australian girls. For him, the frank, outspoken expression of opinion on whatever subject struck the two sisters was at first amusing and refreshing only to a man of his world, but as the acquaintance ripened he felt a very definite interest in the motherless pair, more especially when he found that the aunt to whom they were going in London was an old friend of his father's. Winifred from the first had appealed to him by her serious yet sweet nature; her guardianship of Kitty, which was never relaxed under the most exasperating circumstances, her quiet self-possession and absolute truthfulness made her unlike the women he had previously met. The girls avow their intention of making him their friend, and Winifred, who has an intuitive perception of character, sums him up at his request: "I think you could be frightfully and unreasonably jealous, she said . . . jealous enough to make yourself and other people exceedingly unhappy, especially yourself. . . . You are very proud, too, and if you cared for anybody you would care a very great deal . . . You could be awfully kind. . . and I am just as sure that you could be very cold and cruel too . . . You wouldn't make a very nice enemy." Sir Basil owns to the unqualified truth of this rapid summary of the main points in his character, and he and the two girls become staunch allies in spite of Winifred's plain speaking. Sir Basil exercises a strong influence over the life of one of them, but the story must not be given away here.

* "Irresponsible Kitty." By Curtis York . (John Long. 6s.)

### Notes and Queries.

**REGULATIONS.**

**The Editor is always willing to answer in this column, without any fee, all reasonable questions, as soon as possible.**

**But the following rules must be carefully observed.**

1. **Every communication must be accompanied by the name and address of the writer.**
2. **The question must always bear upon nursing, directly or indirectly.**

**If an answer is required by letter a fee of half-a-crown must be enclosed with the note containing the inquiry.**

*Nursing in Switzerland.*

(164) I am a trained nurse and desire to work in Switzerland. I enclose a letter if you will kindly send it for me to any institution likely to employ me.—*R. P. W.*

You should advertise, and please remember that a penny stamp does not convey a letter to Switzerland.

*Visiting Nurses.*

(165) Is there an Association for the Supply of Visiting Nurses?—*Invalid.*

(166) (1) Where can I write about daily visiting nurses?; (2) and may I have the address of the Norland Institute?—*Nurse W.*

Write to the Marylebone Daily Visiting Nursing Association, 126 Seymour Place, Marylebone, W., and to the Ada Lewis Nursing Institute, 62 Oxford Terrace, W. The Norland Institute is at 10 Pembridge Square, W.

*Temperature.*

(167) I am nursing a patient suffering from valvular disease of the heart, and whose right lung has not quite cleared up after pneumonia. Her evening temperature is never under 100° F., and it is often 101° F. and higher, when taken in the mouth. But when taken in the axilla it is never over normal. Can you kindly offer any explanation of this difference? Could bad teeth have anything to do with it? My patient is very pale, but not emaciated.—*Nurse W.*

As a rule the temperature of the mouth is slightly higher than that of the axilla, there are many causes which might account for the difference you quote; but without seeing the patient it would be impossible to give an opinion.

*Training in several Hospitals.*

(168) I should be glad to know if certificates granted for two years' training in a women's and children's hospital, for one year in a men's surgical hospital, and for eighteen months' work in a fever hospital, will be recognised as equal to one for three years in a general hospital?—*P. M.*

They would not be regarded as equal. It is a pity that you have not kept to one hospital, and thus gained a full and satisfactory certificate.

*Three Months' Training.*

(169) I should be glad if you could tell me of any hospital (in London, if possible) where I could get three months' training as a nurse. I have been matron in a school for two years, and am told that I could obtain a more remunerative post if I could have this training. I am not in a position to pay the fee which I know some hospitals require.—*R. M. F.*

No hospital will receive probationers for three months without a fee, as during that time the person so received is of no practical use to the institution. Three months' training is of no value, and would simply be time wasted.

*Poor-law Training School.*

(170) Will you kindly tell me if Edmonton Poor-law Infirmary is a good training school for nurses, and if the certificate would enable me afterwards to take the post of superintendent nurse?—*Snowdrop.*

Yes. It is a recognised school in which a full course of three years is given in addition to midwifery, and the average number of beds occupied is 424.

**Handbooks for Nurses.**

| | Post Free. |
|---|---|
| "A Handbook for Nurses." (Dr. J. K. Watson.) ... | 5s. 4d. |
| "Nurses' Pronouncing Dictionary of Medical Terms" | 2s. 0d |
| "Art of Massage." (Creighton Hale.) ... ... ... | 6s. 0d. |
| "Surgical Bandaging and Dressings." (Johnson Smith.) ... ... ... ... ... ... ... ... ... | 2s. 0d. |
| "Hints on Tropical Fevers." (Sister Pollard.) ... | 1s. 8d. |

Of all booksellers or of The Scientific Press, Limited, 28 & 29 Southampton Street, Strand, London, W.C.

### For Reading to the Sick.

THE WAY OF PEACE.

There is a way of peace that leads
Through bordered fields and quiet meads;
Those greenest meadows shepherds keep,
Abiding mid their watered sheep.

Beyond earth's changeful fashioning,
Beyond the sweep of death's wide wing,
Beyond the last dark fall of shade,
That home of endless light is made.

Oh, thither fain my feet would go;
My lips would sing the song they know,
Who, crowned with joy, to Zion press
Along the path of lowliness:

Until—as fades across the bay
The moon's broad track at break of day,
The shining path by pilgrims trod
Ends in full presence of their God.

*From the Inner Life.*

"Blessed are they who have the home-longing, for they shall go home."

Blessed, indeed, is the holy rest of Paradise, the home of peace. "But who are they that shall go?" who are they that shall reach those calm and blessed shores? Those who even in this life have longed for home, who have longed for it not only in sorrow but in joy, who have felt how sad and full for forebodings is even the best of happiness on earth, how many shadows enter the brightest home . . . these, if they have sought the completeness of heaven to make up for their ever-growing needs on earth, if they have ceased to trust in the outward life and restless happiness of this world, and learned to cling more and more in thought to the calm communion of saints, these shall go home.

Those, again, who mourn the imperfections and sorrows of mankind, to whom the sin and misery of earth are as an ever-increasing grief, a haunting sadness in the daytime overshadowing the beauty and brightness of the world—who bear the burdens of others in a strength which is not their own, but yet are well-nigh failing beneath their weight—these shall indeed go home, to the bosom of their Lord, to learn from Him to unravel the mystery of sorrow which He fathomed so deeply Himself in the days of His sojourn upon earth.

There is room, too, in Paradise for "those who on earth have nobly failed"; for the "unfinished souls" whose dreams and aspirations could never be realised here, but who in that waiting-time of rest may perchance gather fresh energies for future work, and learn to know the meaning of the soul-inspiring words: "His servants shall serve Him, for they shall see His face."—*M. E. Townsend.*

A little while,
A little while, and we shall stand without
No more, to hear His Voice; but enter in
With joy unspeakable to see His Face.

## Figures and Tables

**Fig. 8. f1:**
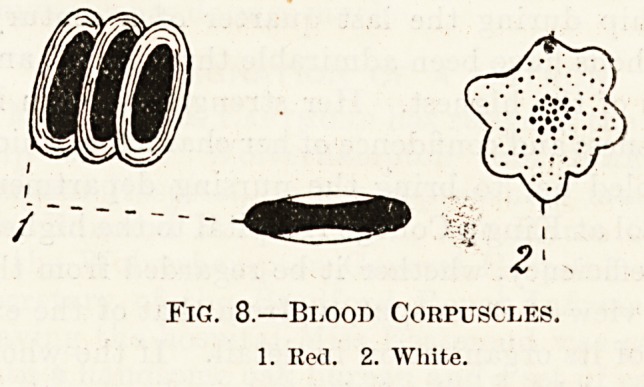


**Figure f2:**
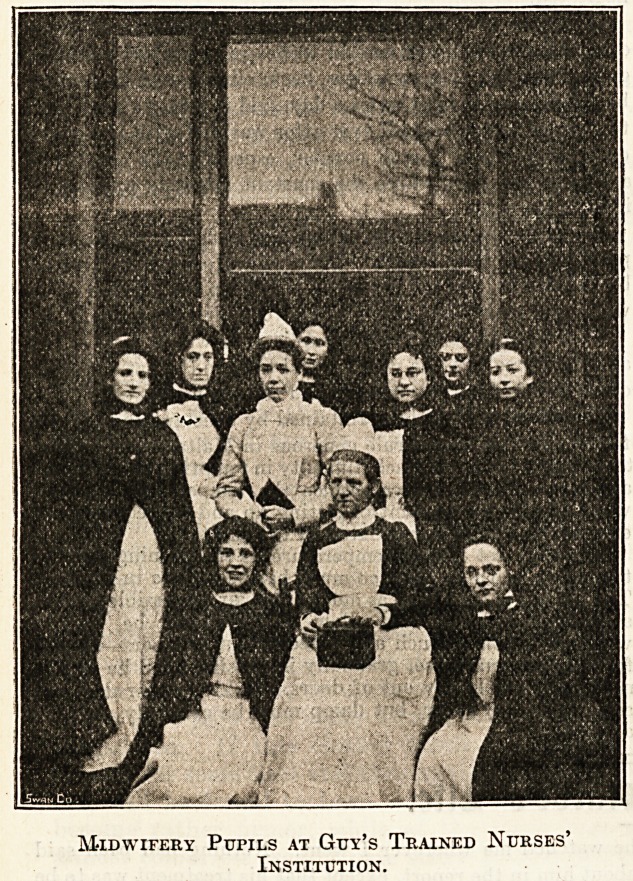


**Figure f3:**
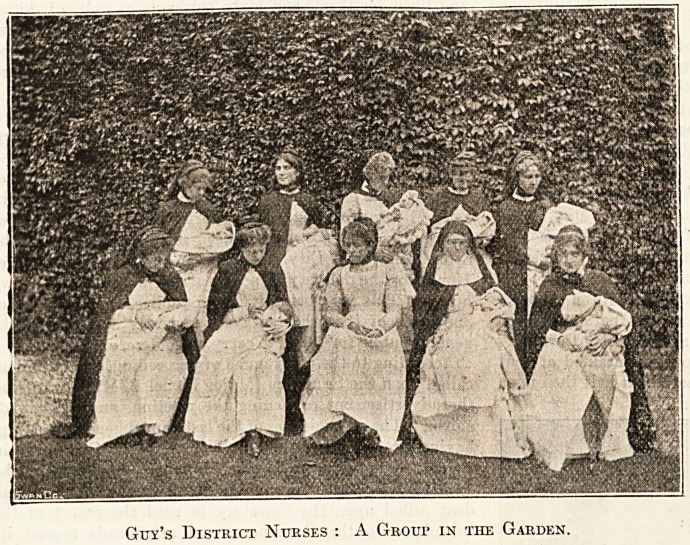


**Figure f4:**
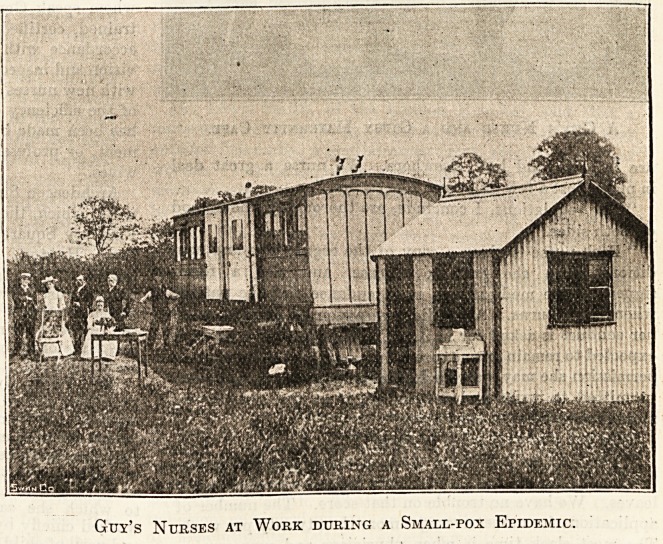


**Figure f5:**